# Hybrid deep learning and optimization-based land use and land cover classification for advancing sustainable agriculture in Najran city, Saudi Arabia

**DOI:** 10.1038/s41598-025-25908-2

**Published:** 2025-11-25

**Authors:** Aisha M. Mashraqi, Eman A. Alshari, Hanan T. Halawani, Ebrahim Mohammed Senan, Yousef Asiri, Bander Mohamd Alowadhi

**Affiliations:** 1 Department of computer science, College of Computer Science and Information Systems, Najran University, Najran, 61441 Saudi Arabia; 2 Department of Artificial Intelligence, Faculty of Computer Science and Information Technology, Al-Razi University, Sana’a, Yemen; 3 Computer Science and Information Technology Department, Thamar University, Dhamar, Yemen; 4 Department of Computer Science, College of Applied Sciences, Hajjah University, Hajjah, Yemen; 5 Technical College of Telecommunications and Information, Jeddah University, Jeddah, Saudi Arabia

**Keywords:** CNN, Land changes classification, Hybrid features, RF, Remote sensing, Najran city, LULC, Engineering, Environmental sciences, Mathematics and computing

## Abstract

In arid regions, land-use/land-cover (LULC) mapping, in central ways, plays a significant role in the sustainability of agriculture. The paper builds on a streamlined hybrid learning system that can categorize the terrain in the Najran, Saudi Arabia, based on 2023 Landsat-8 images to identify indicators of sustainable land use and to guide decisions on the issue. Ten CNN-Random Forest variants were tested; to highlight agronomically informative features, the redundancy of features was minimized with the help of the Ant Colony Optimization. The top models were the ones with high, measured accuracy: VGG19-RF (97.56 overall accuracy, 9726), GoogleNet-RF (96.15), DenseNet121-RF (92.39) and ResNet152-RF (92.26). Class-based area statistics show the presence of built-up area at approximately 29–33%, vegetation area at approximately 14–25%, bare ground at approximately 9–22%, and water area at approximately 9–22%, which reflect the urban growth and development and pressures on developed and irrigated lands. Best models also had a precision/recall/F1 9699% showing dependable separation of agronomic classes. These measured outputs can be converted into operational sustainability indicators: vegetation and bare-soil area to inform crop rotation, soil-cover, and erosion management; built-up encroachment measures to safeguard agricultural buffers; water-body delineation measures to prioritize irrigation efficiency and groundwater recharge areas. The proposed hybrid model is more accurate and interpretable than single-architecture baselines, which provides an extendable avenue toward commonplace LULC monitoring, agricultural risk screening, and policy tracking within the framework of Saudi Vision 2030. The framework can easily be applied to other semi-arid regions in which sustainable production relies on accurate and periodically updated spatial information on the land.

## Introduction

 Remote sensing and geospatial technologies have become irreplaceable for analyzing LULC dynamics that shape environmental and socio-economic systems. Recent satellite missions, including Landsat-8 and Sentinel-2, provide a continuous stream of high-resolution imagery depicting intricate patterns on Earth’s surface for researchers^1^. Manual interpretation, maximum likelihood, and rule-based algorithms, which had been traditionally used in classification methods, have served as the basis of earlier research but have become analytically limited as data volume and complexity have grown. In many instances, strategies fail to capture the detailed spectral variations and temporal changes that characterize heterogeneous landscapes, especially in arid and semi-arid settings such as Najran, Saudi Arabia^2^. Deep learning, as a subset of artificial intelligence, has transformed image classification by enabling models to learn hierarchical representations in an unsupervised way. CNNs are already recognized for their groundbreaking performance across many applications^3^, from object detection to medical imaging, and their popularity in LULC classification is now evolving^4^. Despite those developments, single-architecture CNN models are limited in their ability to generalize across mixed land types and depend on large, labelled datasets. The limitation underscores the need for hybrid deep-learning systems to integrate the complementary capabilities of diverse architectures and combine optimization algorithms to achieve strong performance in complex geospatial environments^5^.

The analytical capacity of single- and traditional models is lagging behind the rapidly evolving field of Earth observation data. Some important contributing factors to high levels of spectral confusion in semi-arid regions, such as soil brightness, sparse vegetation cover, or rough surfaces, lead to misclassification and misinterpretation of soil-use change. Traditional machine learning classifiers, such as SVMs^6^ and RFs, are also heavily reliant on handcrafted features^7^. In contrast, CNN-only practices can be powerful but also risk overfitting or missing important contextual information^8^.

Past studies^9^ to^25^ used single-model structures or statistical classifiers, with moderate accuracy, but they were unable to scale and adapt to space^9^. In addition, few studies have used deep feature extraction and optimization-based feature selection to eliminate redundancy and reduce computational burden^10^. This has created a gap in the field of deep learning regarding the practical implementation of the technology for accurate LULC mapping in Saudi Arabia, where environmental monitoring is a component of national policies, including Vision 2030 and the Green Saudi Initiative^11^.

Based on this, the overall research question is the lack of a hybrid, optimization-enhanced deep learning architecture that would be useful in classifying and detecting land-use and land-cover changes in data-rich, environmentally heterogeneous areas. This gap will be addressed to improve classification accuracy, minimize computational inefficiency, and enhance decision-making^12^.

The general aim of the research is to develop and validate an enhanced hybrid deep learning model for training LULC classification on Landsat-8 imagery over Najran, achieving effective and accurate results. Four objectives guide the study:

- Model Development: Develop and apply ten hybrid deep-learning systems that combine convolutional networks (VGG19, ResNet101, DenseNet169) with a Random Forest classifier to classify LULC in semi-arid environments.
- Feature Optimization: Use the Ant Colony Optimization (ACO) algorithm to select the most applicable spectral-spatial features and remove redundancy, thereby maximizing model generalization and minimizing computational complexity.
- Performance Evaluation: Compare the classification accuracy of hybrid architectures to that of traditional machine learning and single-CNN models using standard statistical measures, such as Overall Accuracy, Kappa Coefficient, Precision, Recall, and F1-Score.
- Applied Impact: Demonstrate the practical usefulness of the optimized hybrid model in contributing to sustainable land-use planning, agricultural monitoring, and environmental policy in line with the objectives of Saudi Vision 2030.

This study contributes significantly to the current literature on remote sensing and artificial intelligence, both methodologically and practically. Methodologically, this study is among the first to introduce a three-layer hybrid model that combines CNN-based feature extraction, cluster-based classification, and heuristic optimization, a novel approach, especially in the Najran region. CNNs, random forests, and ACO form a unified model that captures spectral and spatial dependencies with minimal noise and computational cost. The comparative evaluation of 10 hybrid designs provides valuable insights into how architectural diversity and optimization strategies affect classification performance in abandoned areas.

Applied, the research offers a repeatable analytical framework for extensive LULC charting and observation. The suggested framework is beneficial to policymakers and planners, as it can generate more precise, time-consistent land-cover maps, which are necessary to achieve agricultural sustainability, manage urban growth, and distribute resources. The strategy is also in line with the national environmental goals and provides a practical way to achieve accurate land management nationwide through Vision 2030.

The remainder of the paper is structured as follows: Sect. 2 presents the literature review, and Sect. 3 describes the methodology, including data acquisition, preprocessing techniques, and the proposed architecture. Section 4 will compare the research findings and the system’s performance evaluation with previous studies. Section 5 gives the discussion, and the conclusion is presented in Sect. 6.

## Related work

This section will display a detailed of previous research relevant to this study:

^13^ evaluated changes in LULC in Selangor, Malaysia. From 1991 to 2021, satellite imagery was analyzed using support vector machine (SVM) classification in ArcGIS to generate LULC maps. Future trends were also predicted^14^. examined the interconnections within the Kamrup Metropolitan District, Northeast India, spanning 22 years (2000–2022) and predicted potential outcomes until 2032. Notable changes, like substantial urban expansion and reduced cultivated land, are identified using a precise supervised machine learning algorithm for LULC analysis. Simultaneously, there is a gradual increase in Land Surface Temperature (LST), emphasizing the growing Urban Heat Island (UHI) effect^15^. employed remote sensing and Geographic Information System (GIS) methods, specifically Maximum Likelihood Classification (MLC), to analyze LULC changes over 40 years in the Sahiwal District. Additionally, 120 questionnaires were distributed to local farmers to establish correlations between climate variations and the Normalized Difference Vegetation Index (NDVI). LULC maps for 1981, 2001, and 2021 were generated using MLC, and a regression analysis was conducted to identify the relationship between temperature and vegetation cover in the research area^16^. focused on the YRB, using the Patch Landscape Upscaling Simulation model (PLUS) and the habitat quality model to analyze landscape patterns and habitat quality evolution in the YRB under four scenarios: natural development, farmland protection, urban development, and ecological protection, from the past to 2030^17^. concentrated on classifying Sentinel-2 data based on Land Use, Land Use Change, and Forestry (LULUCF) criteria. The classification is conducted on the Google Earth Engine (GEE), utilizing the RF method. The study evaluates these methods in specific territorial regions (two Czech NUTS 2 units) using data from 2018^18^. introduced an algorithm utilizing Landsat time-series data to assess changes in LULC. The RF classifier, a robust method, was employed in the GEE using Landsat 5, 7, and 8 imagery from 1985 to 2019. The study evaluates the pan-sharpening algorithm’s impact on Landsat bands and explores different image compositions for generating a high-quality LULC map. Additionally, the study examines the influence of various image compositions, spectral indices, and auxiliary data like digital elevation model (DEM) and LST on final classification accuracy^19^. conducted a comparative analysis and accuracy evaluation of Google’s Dynamic World (DW), ESA’s World Cover (WC), and Esri’s Land Cover (Esri) products for the first time. They aimed to guide the future adoption and use of these maps. In the 2023 data, the three global LULC maps exhibit substantial spatial agreement in estimating water, built area, trees, and crop LULC classes. However, WC tends to overestimate grass cover, Esri shows a bias towards shrub and scrub cover, and DW leans towards snow and ice^20^. have assessed the LULC classification performance of ArcGIS Pro and Google Earth Engine, employing various satellite datasets (Landsat, Sentinel, and Planet) in a Charlottetown, Canada case study. ArcGIS Pro used SVM, maximum likelihood, and RF/random tree (RF/RT) classifiers for LULC mapping from 2017 to 2021. Google Earth Engine utilizes SVM, RF/RT, minimum distance (MD), and classification and regression tree (CART) classifiers for the same period^21^. sought to create innovative approaches for automated LULC classification in the Amazon basin, particularly in Brazil, utilizing labels from the MapBiomas project with twelve classes. The research explores diverse fusion methods for combining multi-spectral Sentinel-2 data and synthetic aperture radar Sentinel-1 time series from 2018 in a cloud-prone environment^22^. assessed the suitability of Landsat time series temporal segmentation and RF classifiers for mapping LULC changes, and forest disturbances in Vietnam. The LandTrendr algorithm extracted essential features related to LULCC transitions and forest disturbances from annual Landsat time series data. Individual RF models were created for land use/land cover classification and forest disturbance detection within each segment. The study identified and analyzed concurrent LULCC and forest disturbances from 1988 to 2019^23^. have assessed alterations in land use/land cover from 1991 to 2022 in the Upper Omo–Gibe River basin. The CA-ANN model is utilized to forecast future changes. Landsat-5 TM (1991, 1997, 2004), Landsat-7 ETM+ (2010), and Landsat-8 (OLI) (2016, 2022) were obtained from the USGS Earth Explorer Data Center. LULC classification employed an RF machine learning algorithm^24^. have used a fusion modethat uniquely integrates labels from five directional images (north, south, east, west, and down) to assign brands to image sets. Their model, 2916 GLOBE images, was labeled for land cover classes with minimal human annotation. Validation demonstrated a 90.97% label accuracy, showcasing the potential of our fusion model for efficiently marking extensive land cover databases based on RGB images^25^. presented research that enhances remote sensing knowledge by assessing various Sentinel-2 bands and the adaptability of well-tuned CNNs for semi-arid LULC classification. The study involved training a CNN model in Gujranwala City, Pakistan, and applying the pre-trained model to map LULC in two other semi-arid locations (Lahore and Faisalabad cities).

In brief, this literature review section highlights researchers’ use of remote sensing data for classifying agricultural land. Our examination exposes a dearth of studies employing CNN or hybrid methods for crop and vegetation land classification, a gap evident upon reviewing prior research. This study makes a distinct contribution by addressing this identified gap.

A synthesis of relevant literature is provided in Table 1, which contrasts the methodologies, key findings, and limitations of prior studies with the corresponding methodological advances proposed in this work.

**Table 1:** Summary of related literature on LULC classification, highlighting methodological limitations and the positioning of the present study.

Study (Region & Period)	Primary Data & Platform	Core Methodology	Main Findings/Outputs	Reported/Apparent Limitations	Distinctive Contribution of the Present Work
^13^Selangor, Malaysia (1991–2021)	Landsat; ArcGIS	SVM; trend projection	Multi-decadal LULC maps; future scenarios	Hand-crafted features; limited spatial feature learning	Ten CNN backbones + RF with a comparative benchmark under an identical data regime.
^14^Kamrup, India (2000–2032)	Multispectral; supervised ML	Time-series LULC; UHI–LST analysis	Urban growth; cultivated land decline; LST relations	No deep spatial features; transferability untested	CNN→ACO→RF pipeline yielding higher accuracy and detailed class-wise metrics for agro-monitoring.
^15^Sahiwal, Pakistan (1981–2021)	Landsat; GIS; NDVI + surveys	MLC; climate regression	LULC change; NDVI–climate links	Parametric classifier; sensitive to class heterogeneity	RF on deep CNN features improves non-linear class separation over traditional MLC.
^16^YRB, China (Historic–2030)	Scenario models (PLUS)	Landscape simulation under policy scenarios	Patterns and habitat quality trajectories	Not an image-classification method; focused on scenario inference	Classification-centric approach (Landsat-8, 2023) with pixel-level accuracy suited for agricultural indicators.
^17^Czech Republic (2018)	Sentinel-2; GEE	RF on spectral/indices	Single-year LULUCF map; regional evaluation	Relies on manually engineered indices; single model type	Augments RF with deep CNN embeddings, moving beyond manual feature design.
^18^Multi-region (1985–2019)	Landsat 5/7/8; GEE	RF with indices/DEM, composites	Impact of composites/auxiliary data on accuracy	Manual feature engineering required	ACO automatically selects discriminative feature subsets from CNN outputs before RF classification.
^19^Global (2023)	DW, WC, Esri global products	Cross-product benchmarking	Agreement and class biases across global maps	Known class biases; not locally tuned	Site-optimized model for Najran with OA up to 97.56%, κ ≈ 0.97–0.98, tailored to the arid context.
^20^Charlottetown, Canada (2017–2021)	Multi-sensor; ArcGIS & GEE	SVM, RF, MD, CART comparison	Tool and classifier performance contrasts	Tool-dependent; no deep feature fusion	Systematic hybrid CNN–RF evaluation (10 backbones) with ACO, demonstrating consistent gains.
^21^Amazon Basin, Brazil (2018)	Sentinel-1/2; MapBiomas labels	Optical–SAR fusion for automated LULC	12-class mapping in cloud-prone settings	Fusion complexity; limited deep-hybrid analysis	Focus on arid/semi-arid optics (Landsat-8) with a streamlined hybrid deep + RF approach.
^22^Vietnam (1988–2019)	Landsat time series	LandTrendr + RF	LULCC and forest disturbance timelines	Emphasis on temporal breaks over spatial features	High-fidelity spatial classification for a key timestamp using a CNN→ACO→RF pipeline.
^23^Upper Omo–Gibe, Ethiopia (1991–2022)	Landsat; USGS Earth Explorer	RF; CA-ANN forecasting	Historical change and future projections	No deep feature extraction; arid region performance unvalidated	Semi-arid-aware design; CNN features reduce soil–urban–vegetation spectral confusion.
^24^GLOBE multi-view RGB	Five-view RGB image sets	Label fusion with minimal annotation	~ 90.97% label accuracy on non-satellite images	Small RGB dataset; not comparable to satellite LULC	Uses standard satellite imagery (Landsat-8) with ten CNNs + RF for operational-grade mapping.
^25^Semi-arid Pakistan (Cross-city)	Sentinel-2	Single tuned CNN; transfer learning	Demonstrated CNN feasibility for semi-arid LULC	Single architecture; no hybrid comparison or feature pruning	Comprehensive benchmark of 10 CNN-RF hybrids with ACO; top model VGG19-RF achieved OA 97.56%.

## Methodology

**Fig. 1:**
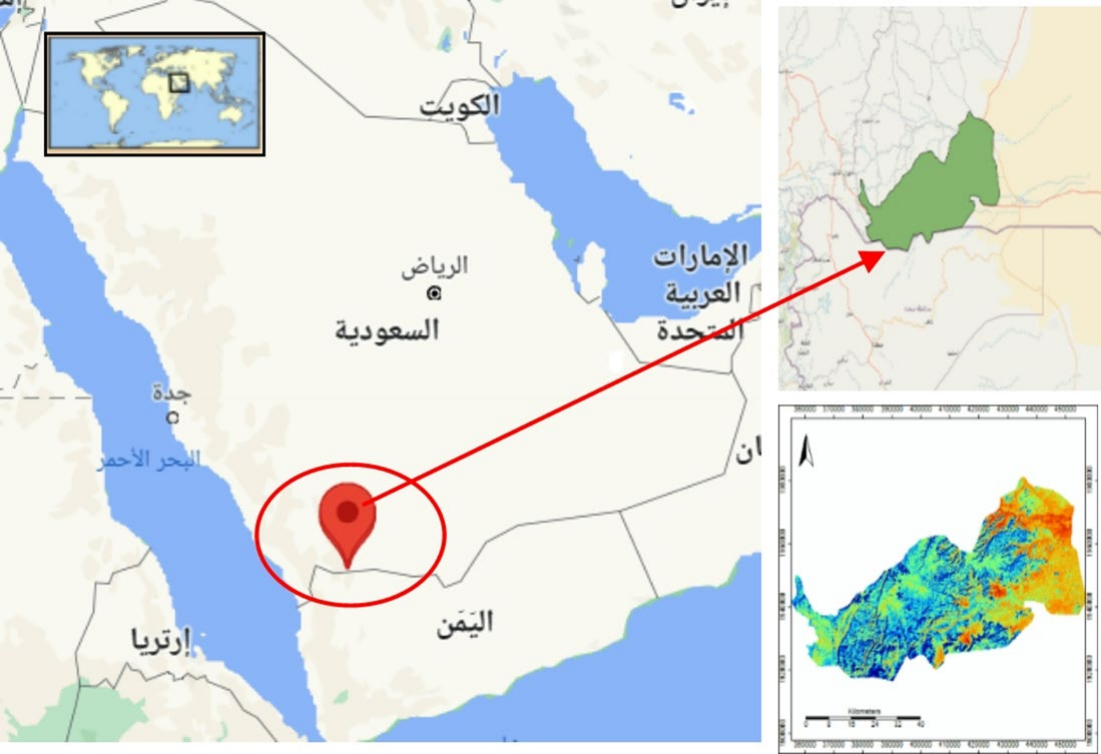
Study location case study.

Najran is a pivotal city and serves as the administrative hub for the Najran province, Fig. 1. It has undergone notable demographic expansion and is acknowledged as a swiftly growing urban center within the kingdom. The exact geographical coordinates of Najran City are 17.565604 degrees North latitude and 44.228944 degrees East longitude, presented in GPS format as Latitude: 17° 33’ 56.1744’’ N and Longitude: 44° 13’ 44.1984’’ E^26^.

### Data sources

**Fig. 2:**
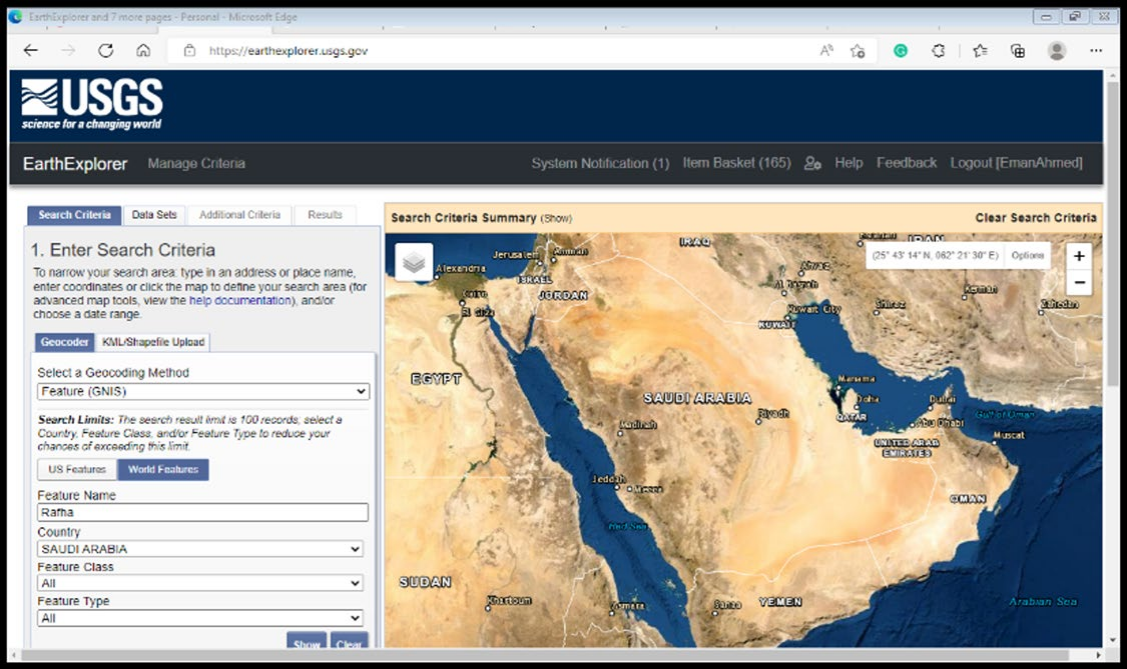
Dataset retrieval from USGS.

The Najran city map in Saudi Arabia was developed using 2023 imagery from the Landsat 8 satellite. Managed by the USGS and NASA, Landsat 8 offers high-resolution, multispectral images worldwide. These images, collected across various spectral bands, are crucial for analyzing land cover, vegetation health, and urban development. Geospatial processing and map creation were performed using SAGA GIS (v. 2.3.2) within the QGIS Geographic Information System (v. 3.6; Open-Source Geospatial Foundation, http://qgis.org) Fig 2.

The initial step of the experiment involves obtaining essential data. It includes securing Landsat 8 satellite imagery from 2010 to 2023, along with the corresponding NDVI data. The NDVI data is sourced from the United States Geological Survey (USGS) or the European Space Agency (ESA). A foundational map is generated using survey images of the SOI toposheet at a 1:50000 scale. The Landsat 8 sensor for 2023 facilitates the calibration and comparison of land, as indicated in Table 2. Typically, the visuals consist of maps depicting various scales, dates, and times.

**Table 2:** Landsat 8 satellite imagery and database specifications for LULC of Najran.

Date Acquired	Landsat 8 Satellite
Sensor	The sensor is an OLI and thermal Infrared Sensor
Satellite	Landsat8 Satellite
Resolution	30 m
Size of database	16 images
The time of the season	June

The satellite collects spectral reflectance data through eleven bands, covering diverse spectrum wavelengths. Seven bands specifically target the visible, near-infrared, and shortwave infrared segments, providing a spatial resolution of 30 m within a 185-kilometer swath. These selected bands were incorporated into the land use dataset, compiled from 50 scenes obtained in Najran in 2023. Each image in the dataset comprises pixels with dimensions of 6441 × 6441^5^, as outlined in Table 3.

**Table 3:** Landsat-8 OLI spectral bands used for LULC mapping in Najran (2023).

Num	Types and Components of Bands
1	Band 1 (0.43–0.45 μm)
2	Band 2 (0.450–0.51 μm)
3	Band 3 (0.53–0.59 μm)
4	Band 4 (0.64–0.67 μm)
5	Band 5 (0.85–0.88 μm)
6	Band 6 (1.57–1.65 μm)
7	Band 7 (2.11–2.29 μm)

### Data preprocessing

**Fig. 3:**
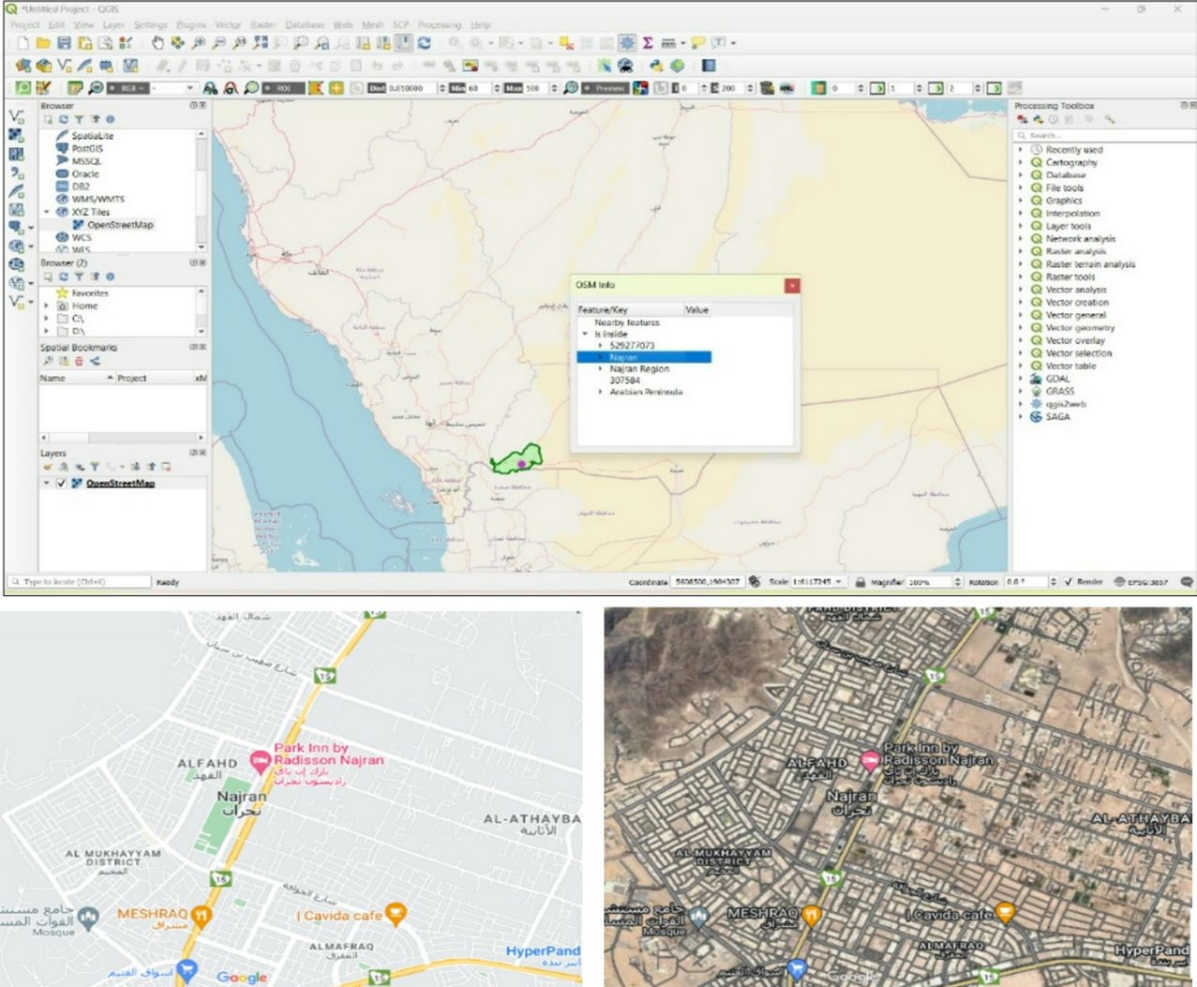
Location Najran in QGIS software, Google Earth, and Google map.

The preprocessing stage in Land Use Change involves several crucial steps. The initial step, Clip-ping Map, defines specific areas on the world map and cuts the map accordingly. This stage is vital to the overall Land Use Change process. Image Registration and Image Enhancement are subsequent phases in preprocessing remote sensing data. Image registration is performed automatically or manually using SOMINFO, which is installed in QGIS plugins. The location map is then displayed in Open Layer Map for automatic image registration. Following this, Image Enhancement is carried out to improve the resolution of retrieved images. The subsequent stages involve Geometric, Radiometric, and Radiometric correction, specifically to Campsite bands, particularly band 432. The overall process includes the essential tasks of clipping and registering images. The coordinate reference system is critical for defining and cutting, and this process includes studying the exact location of the case study, as shown in Fig. 3 on Google Maps. The information is divided into images in either WGS84 or WGS84/UTM, and data downloaded from satellites under remote sensing technology is precisely identified in QGIS.

**Fig. 4:**
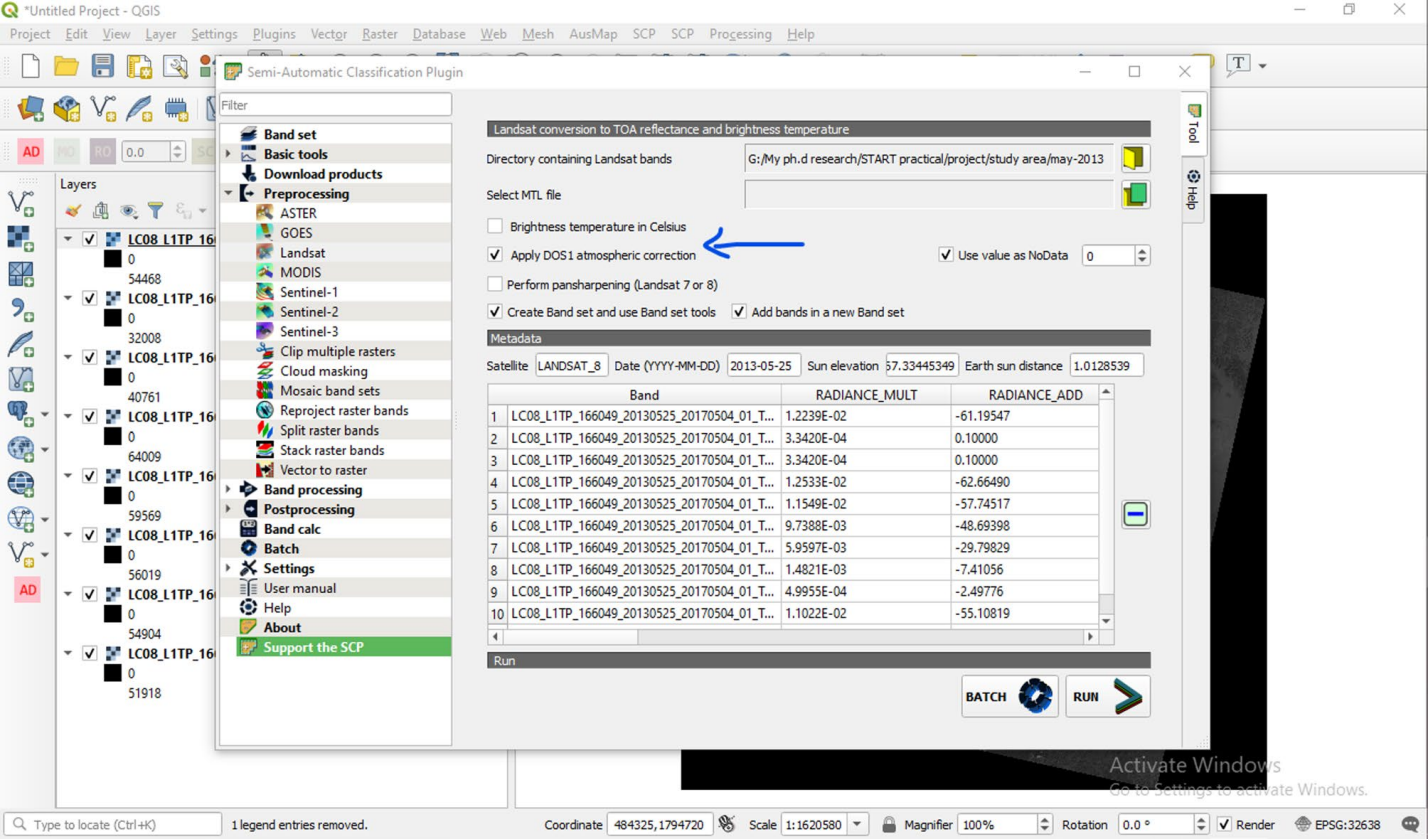
Processing of Radiometric Corrections.

Geometric correction in remote sensing involves aligning data with a specific map’s scale and projection^27^. Registration matches the coordinate systems of two images taken at the exact location, while geometric correction adjusts pixel locations without changing their radiant reflectance. Placing pixels in inaccurate map coordinates is called geometric rectification. If the Open Layer Map displays differently, manual image registration is necessary, involving steps like toposheet georeferencing and image registration. Georeferencing a toposheet establishes a mathematical connection between image and spatial coordinate systems. Image registration overlays multiple images of the same scene, connecting georeferenced base imagery with satellite image coordinates. Radiometric correction aims to enhance the accuracy of brightness values in remote-sensing photos. It processes digital images to reduce inaccuracies in brightness values. These correction and geometric adjustments improve product accuracy by addressing variations in correction orders. Radiometric calibration and atmospheric correction re-calibrate pixel radiance to prevent errors. Geometric correction eliminates distortion through registration, local incident angle corrections, and knowledge of geodetic coordinates^28^. The combined process of geometric and radiometric corrections is depicted in Fig. 4.

Image Enhancement involves two main processes: noise removal and radiometric corrections, specifically applied to satellite images. The goal is to enhance these images for improved classification and to rectify any degradation, ensuring a more accurate representation of the original scene. These operations, collectively known as preprocessing, are crucial as they occur before the data is utilized for a specific purpose. Preprocessing is essential to address shortcomings and eliminate faults in remote sensing data, enhancing radiometric and geometric accuracy during acquisition, scanning, and transmission. Radiometric correction, a crucial aspect of image enhancement, considers environmental factors, while noise reduction becomes necessary due to information and recording procedures limitations^7^.

### Techniques used in this study

This section will provide differences in the techniques implemented for this work. Understanding the nuances of these CNN architectures is paramount for researchers, practitioners, and enthusiasts alike, as it enriches our knowledge of deep learning methodologies and enables informed choices when selecting models for specific applications by examining each model’s strengths and weaknesses. It strives to contribute to a deeper comprehension of the ever-expanding landscape of CNN and its implications for advancing the frontiers of computer vision^29^.

For Najran City, the choice of a feature extraction method depends on factors like the availability of computational resources, the complexity of the land change patterns, and the desired trade-off between accuracy and efficiency. Conducting experiments with these architectures on the specific dataset from Najran City would be essential to determine their performance for land change classification in that particular region. Feature extraction is a critical step in land change classification, as it involves transforming raw input data from satellite imagery into a set of representative features used for category. The convolutional layers are responsible for feature extraction by applying convolution operations to the input data^30^. A filter is a small matrix used to input data, capturing specific features like edges, textures, or patterns, the dimensions of the filter, etc. Convolutional layers produce feature maps that represent the presence of particular features in the input. This work’s pooling layers down sample feature maps reduce spatial dimensions and computational complexity. This study used ACO to reduce components, keep essential features, and remove repeating elements. There is max pooling (retains max value) and average pooling (contains average value)^31^.

#### Rationale for model selection and algorithmic design

Najran’s semi-arid terrain, bright soils, sparse vegetation, and mixed urban–agriculture edges demand representations that capture multi-scale textures, preserve gradient flow in deep hierarchies, and remain stable under limited labels. The hybrid pipeline therefore stacks convolutional backbones for feature extraction, ACO for redundancy-aware feature pruning, and a RF head for variance-reduced decision making.

The ten CNN architectures employed in this study were selected to provide a comprehensive evaluation of diverse deep learning design paradigms. This selection encompasses pioneering models (LeNet-5, AlexNet), very deep networks (VGG19), architectures with advanced connectivity to facilitate gradient flow and feature reuse (ResNet152, DenseNet121), and models optimized for computational efficiency (SqueezeNet, MobileNetV2, ShuffleNet). Furthermore, we included models renowned for multi-scale featre extraction (GoogleNet) and those derived from a systematic design space exploration (RegNet). This strategic diversity ensures a robust comparative analysis, allowing us to identify the architectural features most conducive to accurate land cover classification in the Najran study area.

RF atop frozen embeddings provide non-parametric class boundaries, out-of-bag validation, and resilience to class imbalance, while ACO selects compact, discriminative subsets, curbing overfitting and inference cost^32^.

Table 4 displays the optimized hyperparameters and training metrics for the ten CNN architectures tested in this research. The architectures in this table demonstrate a purposeful architecture-aware tuning strategy that was carefully selected for the challenges present in the Najran terrain. The used optimizer was also intentionally selected, as employed SGD with Nesterov momentum for deeper and more traditional architectures (VGG19 and ResNet152) to ensure stable convergence in more complicated loss landscapes, while Adam was used with more efficient modern networks (MobileNetV2 and ShuffleNet) to accelerate training and still enable us to retain good performance on LULC taxonomy.

Moreover, the learning rates and batch sizes were selected with the motivation of achieving a balance between convergence speed, generalization, and hardware limits. Additionally, the deeper networks, VGG19 and ResNet152, required lower batch sizes (8–16) but with higher learning rates (1e-2) so as to balance the complexity of training and memory usage, which resulted in longer training times, but better final losses (Val Loss: 0.134–0.148), if ultimately better representational performance overall. Meanwhile, the lightweight networks, such as SqueezeNet and ShuffleNet, had larger batch sizes (128) and used more moderate learning rates (1e-3), which resulted in faster training times (25–35 min), but had larger validation losses (0.278–0.361) because there is a clear trade-off between computational efficiency and representational power. It was also nice to see that all of the models reliably converged based on the fact that the training and validation losses remained so close, which provided confidence in the final selected parameters being appropriate for LULC classification of a semi-arid environment^33^.

**Table 4:** Hyperparameter tuning and training metrics for CNN models for Najran terrain classification.

Model	Input Size	Batch Size	Optimizer	Learning Rate	Epochs	Training Time (min)	Train Loss	Val Loss
**LeNet-5**	64 × 64	128	Adam	1.0 × 10⁻³	50	18	0.315	0.368
**AlexNet**	227 × 227	64	SGD + Nesterov	5.0 × 10⁻³	45	45	0.238	0.305
**VGG19**	224 × 224	16	SGD + Nesterov	1.0 × 10⁻²	40	240	0.105	0.148
**GoogleNet**	224 × 224	32	SGD + Nesterov	2.0 × 10⁻³	45	85	0.118	0.162
**ResNet152**	224 × 224	8	SGD + Nesterov	1.0 × 10⁻²	35	180	0.092	0.134
**DenseNet121**	224 × 224	16	SGD + Nesterov	2.0 × 10⁻³	40	95	0.112	0.152
**MobileNetV2**	224 × 224	64	Adam	1.0 × 10⁻³	50	35	0.152	0.198
**ShuffleNet**	224 × 224	128	Adam	2.0 × 10⁻³	50	28	0.305	0.361
**SqueezeNet**	224 × 224	128	Adam	1.0 × 10⁻³	50	25	0.218	0.278
**RegNet**	224 × 224	32	SGD + Nesterov	2.0 × 10⁻³	45	65	0.128	0.175

#### CNN for feature extraction

Feature extraction with convolutional layers is crucial for reducing the dimensionality of input data while retaining important information, especially in image classification of land change tasks^34,35^. CNNs utilize convolutional layers to automatically learn and extract hierarchical features from input images. In the case of classifying land changes in Najran using the 2023 map with ten different algorithms (LeNet5, VGG19, SqueezeNet, RegNet, MobileNetV2, ResNet152, AlexNet, GoogleNet, ShuffleNet, and DenseNet121), the process involves several steps. Initially, the input map is preprocessed to enhance features and normalize pixel values. Subsequently, the preprocessed map is fed into the respective CNN architecture, and the convolutional layers learn spatial hierarchies of features like edges, textures, and patterns. As the network progresses through layers, more complex and abstract features related to land changes in Najran are extracted. The extracted features are flattened and passed through fully connected layers for classification. Each algorithm adapts its architecture, depth, and parameters to capture distinct features, contributing to the overall accuracy and efficiency in classifying land changes in Najran based on the 2023 map. The diversity in architectures, from the simplicity of LeNet5 to the depth of ResNet152 and the efficiency of MobileNetV2, ensures a comprehensive exploration of feature spaces, ultimately enhancing the moels’ ability to discern and classify different land changes^36^.

The CNN’s extracted deep features are employed for subsequent classification analysis. Fundamental to CNNs are convolutional layers, which apply filters to the input image^37^. These filters, typically sized 3 × 3, 5 × 5, or 7 × 7, determine the receptive field, the portion of the input image examined by the filter as in Eq. 10. Smaller filters capture finer details, while larger ones capture more extensive features. The padding adds extra rows and columns of zeros to the input image to maintain spatial dimensions and prevent feature map reduction post-convolution. The stride, controlling the filter’s shift over the input image, dictates how many pixels the filter moves between applications. Following convolution, an activation function, often ReLU, is applied to enable the network to learn intricate relationships among features^35^.

10 $$\:y\left(t\right)=\left(x\text{*}f\right)\left(t\right)=\int\:x\left(a\right)f\left(t-a\right)\:\:da$$

The filter function is represented by *f(t)*, the input image is denoted by *x(t)*, and the resulting output is indicated by *y(t)*.

The purpose of pooling layers is to decrease the size of feature maps generated by convolutional layers. These layers apply a pooling function to specific regions of the feature map, which sample the map by summarizing pixel values in each area. Two primary types of pooling layers are average pooling and max pooling. These layers contribute to the efficiency of CNNs by reducing feature map sizes, mitigating overfitting risks, and extracting more robust image features. Average pooling involves calculating the average value of a set of neighboring pixels, facilitating a reduction in spatial dimensions and computational complexity. For instance, in 2 × 2 average pooling, a single pixel replaces each 2 × 2 block, and its value is the average of the four pixels in the block, as expressed in Eq. 11. On the other hand, max pooling selects the maximum value from a group of neighboring pixels, retaining dominant features and preserving spatial hierarchies, as illustrated in Eq. 12. Similar to average pooling, max pooling also contributes to a reduction in spatial dimensions.

11 $$\:z\left(i;\:j\right)=\frac{1}{{k}^{2}}\sum\:_{m,n=1\dots\:.k}f\left[\left(i-1\right)p+m;\:\left(\:j-1\right)p+n\right]$$

12 $$\:z\left(i;\:j\right)={max}_{m,n=1\dots\:.k}\:f\left[\left(i-1\right)p+m;\:\left(\:j-1\right)p+n\right]$$

Where *f* is the convolutional filter applied to a specific region in the image. The variables *m* and *n* are precise coordinates in the image matrix. The variable *k* represents the total number of pixels in this region, and *p* mean a specific step in the process.

CNNs automatically acquire hierarchical representations of input data by incorporating multiple convolutional and pooling layers. In our study, we utilized CNN models to extract critical features efficiently^38,39^.

#### Ant colony optimization algorithm

The ACO algorithm is crucial in feature reduction to image classification of land change tasks. Feature reduction is essential to enhance the efficiency and performance of classification algorithms by selecting the most relevant features and discarding irrelevant or redundant ones^40^. Inspired by the foraging behavior of ants, ACO is particularly effective in optimization problems. In classifying land changes in Najran using the classified map of 2023, the ACO algorithm employed to optimize the feature selection process for each of the ten specified algorithms (LeNet5, VGG19, SqueezeNet, RegNet, MobileNetV2, ResNet152, AlexNet, GoogleNet, ShuffleNet, and DenseNet121). The ACO algorithm begins by initializing a population of ant solutions, each representing a subset of features. Ants iteratively construct solutions by selecting features based on a probability distribution influenced by the quality of features. The algorithm uses pheromones to update the probabilities dynamically, enabling efficient exploration of the feature space. After several iterations, the algorithm converges to a subset of features that optimally represent the land changes in Najran. The optimized feature set is then fed into the RF algorithms, enhancing their accuracy and efficiency in discerning land-use changes based on the 2023 map.

Starting with pheromone levels (τij) at a modest positive value across all features, an arbitrary quantity of artificial pheromone (τ0) is allocated to each part. In individual ants symbolize prospective subsets of features. As ants navigate the feature space, they make feature selections through probabilistic decisions. The likelihood of ant i selecting feature j is determined by Eq. 1:

1 $$\:{p}_{ij}\text{}=\frac{{\tau\:}_{ij}^{\alpha\:}\left(t\right).\:{\eta\:}_{ij}^{\beta\:}}{{\sum\:}_{k\in\:Features}{\tau\:}_{ik}^{\alpha\:}\left(t\right).\:{\eta\:}_{ik}^{\beta\:}}$$

The parameters α and β govern the impact of pheromone and heuristic information, respectively. η_ij represents the quality of feature j. Ants create a feature subset guided by probabilities determined in step 2. The assessment of each ant’s feature subset involves a fitness function related to detecting changes in land. Pheromone levels are adjusted according to the quality of solutions discovered by the ants, following Eq. 2:

2 $$\:{\tau\:}_{ij}\leftarrow\:(1-{\rho\:)\:.\tau\:}_{ij}\:+\:\sum\:_{Ants}{\varDelta\:\:\tau\:}_{ij}\text{}$$

The rate at which pheromones evaporate is denoted by ρ. The pheromones deposited by ant i on feature j are represented by $\:\sum\:_{Ants}{\varDelta\:\:\tau\:}_{ij}\text{}$. Follow steps 2–5 for a specified number of iterations. After the last iteration, the ultimate feature subset is chosen by evaluating the pheromone levels. The ACO algorithm progressively improves feature subsets by considering the pheromone trails left by the ants^29,31,41^.

The primary rationale for selecting ACO over other metaheuristics (such as Genetic Algorithms, Particle Swarm Optimization, or Simulated Annealing) is its inherent suitability for combinatorial optimization problems, such as feature selection. Our problem involves selecting an optimal subset of features from a large, high-dimensional pool extracted by the CNNs. ACO is inherently designed for such problems, as it mimics the process of ants finding the shortest path to a food source—a direct analogy to finding the most efficient (i.e., most discriminative) path through the feature space.

Relative to alternative used for feature selection (e.g., GA, PSO, SA), ACO offers three practical advantages documented in the literature and leveraged in our pipeline: (i) seamless handling of binary inclusion decisions without encoding/decoding overhead; (ii) the ability to inject domain heuristics (e.g., per-feature relevance from mutual information or out-of-bag importance from Random Forest) directly into the construction probabilities, accelerating convergence toward compact subsets; and (iii) “anytime” performance—useful intermediate subsets emerge early and improve monotonically with additional iterations, which is desirable when compute budgets are finite. From an application standpoint, our downstream classifier (RF) is known to be robust yet sensitive to redundant, collinear inputs; reducing overlap among deep features typically improves generalization and interpretability.

#### Connecting to random forest algorithm

Random Forest is an ensemble learning method that constructs multiple decision trees during training and aggregates their outputs to improve classification accuracy and generalization. The RF classifier mitigates overfitting, reduces variance, and enhances the robustness of predictions by relying on bootstrap aggregation (bagging). CNN models extract high-dimensional spectral features from input images. However, CNNs primarily perform feature extraction, while the final classification stage often benefits from alternative RF classifiers. RF effectively handles high-dimensional feature vectors from CNNs by leveraging multiple decision trees. RF can model complex feature relationships without assuming linearity unlike traditional classifiers such as logistic regression. RF performs well on high-dimensional CNN features by averaging multiple decision tree predictions, thus reducing overfitting. After CNN models extract deep features from Najran map images, these feature vectors are flattened into numerical representations that serve as inputs to the RF classifier. The CNN feature maps are transformed into fixed-length feature vectors to serve as inputs for the RF classifier. The extracted feature vectors are fed into RF, which builds multiple decision trees based on different random subsets of features. The RF classifier aggregates predictions from multiple decision trees to determine the final class label^34^.

### Experimental proposed approaches

**Fig. 5:**
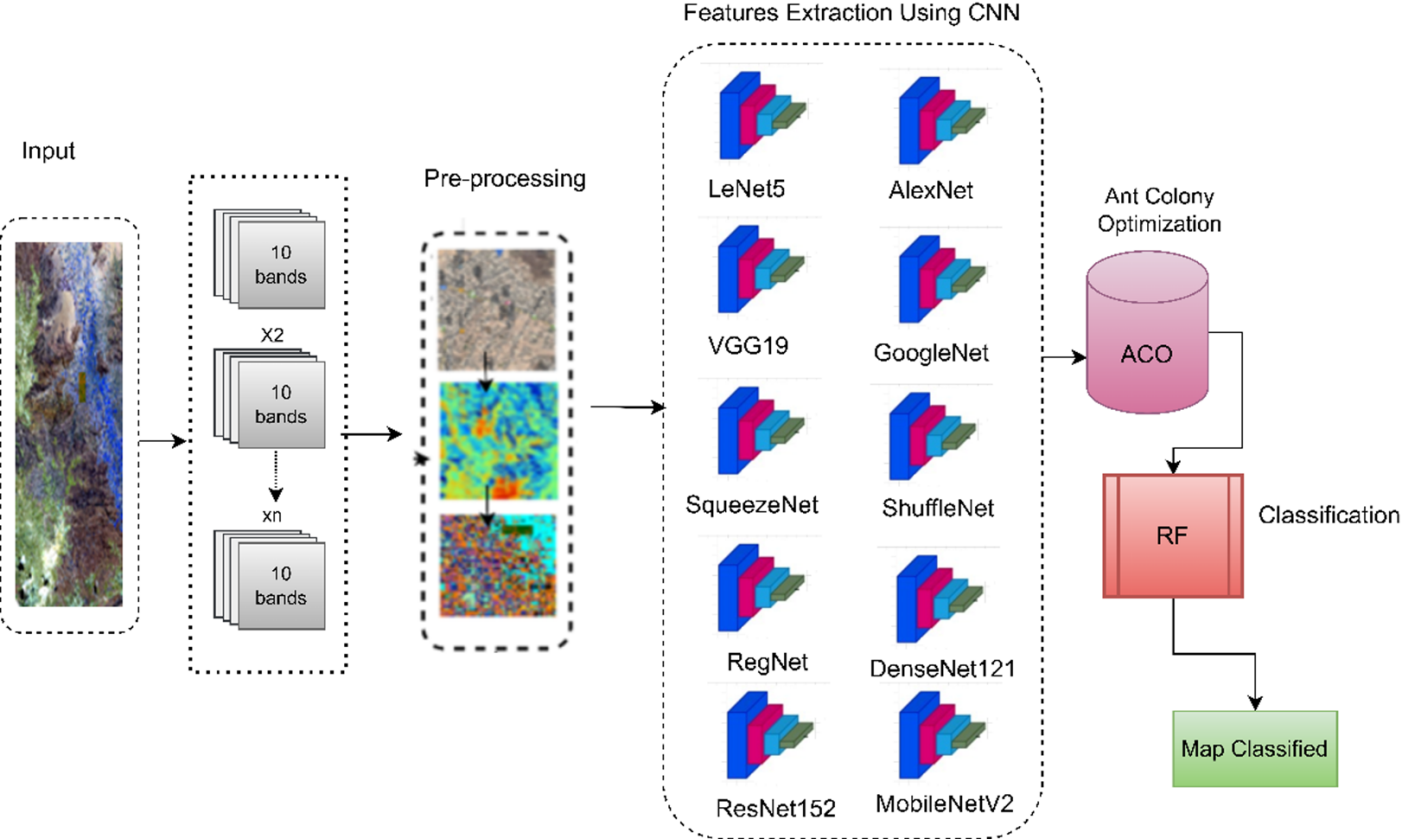
The Proposed Approach Hybrid.

The experimental processes for classifying changes in the land of Najran using the specified ten algorithms begin with the convolutional layer in each model. In the case of LeNet5, VGG19, SqueezeNet, RegNet, MobileNetV2, ResNet152, AlexNet, GoogleNet, ShuffleNet, and DenseNet121, the convolutional layers are responsible for extracting hierarchical features from the input land images. Subsequently, the feature extraction step involves progressively learning abstract representations of the land changes through the network’s architecture, capturing spatial and temporal patterns. Following feature extraction, an ACO algorithm is employed to reduce the dimensionality of the feature vectors, enhancing computational efficiency and retaining crucial information. The reduced features are fed into an RF algorithm, a classifier to categorize the land changes. The RF classifier leverages the collective decision-making of multiple decision trees to improve accuracy. Finally, the classified map for 2023 is generated, providing a comprehensive and accurate depiction of the land changes in Najran based on the outputs of these diverse algorithms. Figure 5 is a summary process of the hybrid proposed approach.

## Results

### Overall accuracy and kappa coefficient

Overall accuracy and the kappa coefficient serve as performance metrics for evaluating the effectiveness of a classification model. Overall accuracy represents the proportion of correctly classified instances among the total cases in the dataset, providing a straightforward measure of correctness. The kappa coefficient, adjusts this by accounting for the expected agreement based on class distributions, making it a more robust measure of classification performance, particularly in imbalanced datasets.

Table 5 presents the accuracy and kappa coefficient of the proposed hybrid approach. The hybrid classifiers include LeNet5-RF, VGG19-RF, SqueezeNet-RF, RegNet-RF, MobileNetV2-RF, ResNet152-RF, AlexNet-RF, GoogleNet-RF, ShuffleNet-RF, and DenseNet121-RF. Among these, VGG19-RF stands out with the highest accuracy of 97.56% and a notable kappa coefficient. GoogleNet-RF also shows impressive results with an accuracy of 96.15% and a kappa coefficient of 0.983502. DenseNet121-RF achieves a high accuracy of 92.39% and a kappa coefficient of 0.973301, while Res-Net152-RF attains an accuracy of 92.26%. These four hybrid classifiers—VGG19-RF, GoogleNet-RF, ResNet152-RF, and DenseNet121-RF—are particularly noteworthy for their exceptional accuracy and strong agreement between predictions. and actual out-comes, making them noteworthy choices for the given evaluation task.

**Table 5:** Overall accuracy & kappa coefficient of hybrid proposed Approach.

NO	A hybrid Classifier	Overall Accuracy %	Kappa Coefficient	Evaluation
1	LeNet5-RF	88.92	0.998203	Moderate
2	VGG19-RF	97.56	0.972608	High
3	SqueezeNet-RF	89.49	0.956202	Moderate
4	RegNet-RF	90.56	0.931802	High
5	MobileNetV2-RF	88.83	0.739504	Moderate
6	ResNet152-RF	92.26	0.914909	High
7	AlexNet-RF	94.26	0.908008	Moderate
8	GoogleNet-RF	96.15	0.983502	High
9	ShuffleNet-RF	86.69	0.739504	Low
10	DenseNet121-RF	92.39	0.973301	High

Overall, the results suggest that different deep learning models coupled with RF algorithm exhibit varying degrees of accuracy in identifying land cover categories. The choice of model significantly impacts the accuracy of land cover mapping, with certain models performing better in specific categories than others. Further analysis and validation are necessary to determine the most suitable model for accurate land cover mapping in a given area.

Table 6, which presents results for Group 1, land use/land cover was classified into five primary classes: Built-up, Vegetation, Water, Bare Soil, and Other Land. These categories were identified based on spectral, textural, and contextual features extracted from satellite imagery using various CNN architectures (LeNet5, VGG19, SqueezeNet, RegNet, and MobileNetV2) integrated with RF classifier. The Built-up class shows a high variation, ranging from 20.02% (RegNet-RF) to 32.75% (SqueezeNet-RF). This variation reflects differences in the ability of each model to capture the spatial density and edge features characteristic of urban structures. Vegetation percentages remain relatively stable, between 14.08% and 25.04%, indicating that vegetation was consistently recognized across models due to its distinctive spectral signature in the near-infrared band. The Water class varies more significantly, especially for RegNet-RF (3.40%), suggesting that some models may have underrepresented water bodies, possibly due to seasonal changes or confusion with shadows or bare soils. In contrast, Bare Soil is highest in RegNet-RF (29.88%), likely due to its sensitivity to low-texture regions. The MobileNetV2-RF (21.64%) also captured bare soil effectively despite being a lightweight model. The Other Land category is notably highest in MobileNetV2-RF (34.25%), which may be attributed to the classification of mixed pixels or transitional areas not clearly matching other categories.

**Table 6:** Group1 area and percentages land changes for proposed Approach.

Models	LeNet5-RF		VGG19-RF		SqueezeNet-RF		RegNet-RF		MobileNetV2 RF	
Classes	Area km²	Area %	Area km²	Area %	Area km²	Area %	Area km²	Area %	Area km²	Area %
Built up	309.9	26.80%	346.34	29.95%	378.59	32.75%	231.49	20.02%	257.3	22.24%
Vegetation	282.73	24.45%	267.11	23.09%	282.36	24.42%	289.59	25.04%	162.86	14.08%
Water	241.89	20.92%	198.28	17.13%	178.3	15.43%	39.31	3.40%	90.06	7.79%
Bare Soil	104.57	9.05%	122.18	10.56%	109.15	9.44%	345.55	29.88%	250.09	21.64%
Other Land	217.06	18.77%	222.24	19.27%	207.75	17.96%	250.21	21.65%	396.84	34.25%
**Total**	**1**,**156.15**	**100%**	**1**,**156.15**	**100%**	**1**,**156.15**	**100%**	**1**,**156.15**	**100%**	**1**,**156.15**	**100%**

**Fig. 6:**
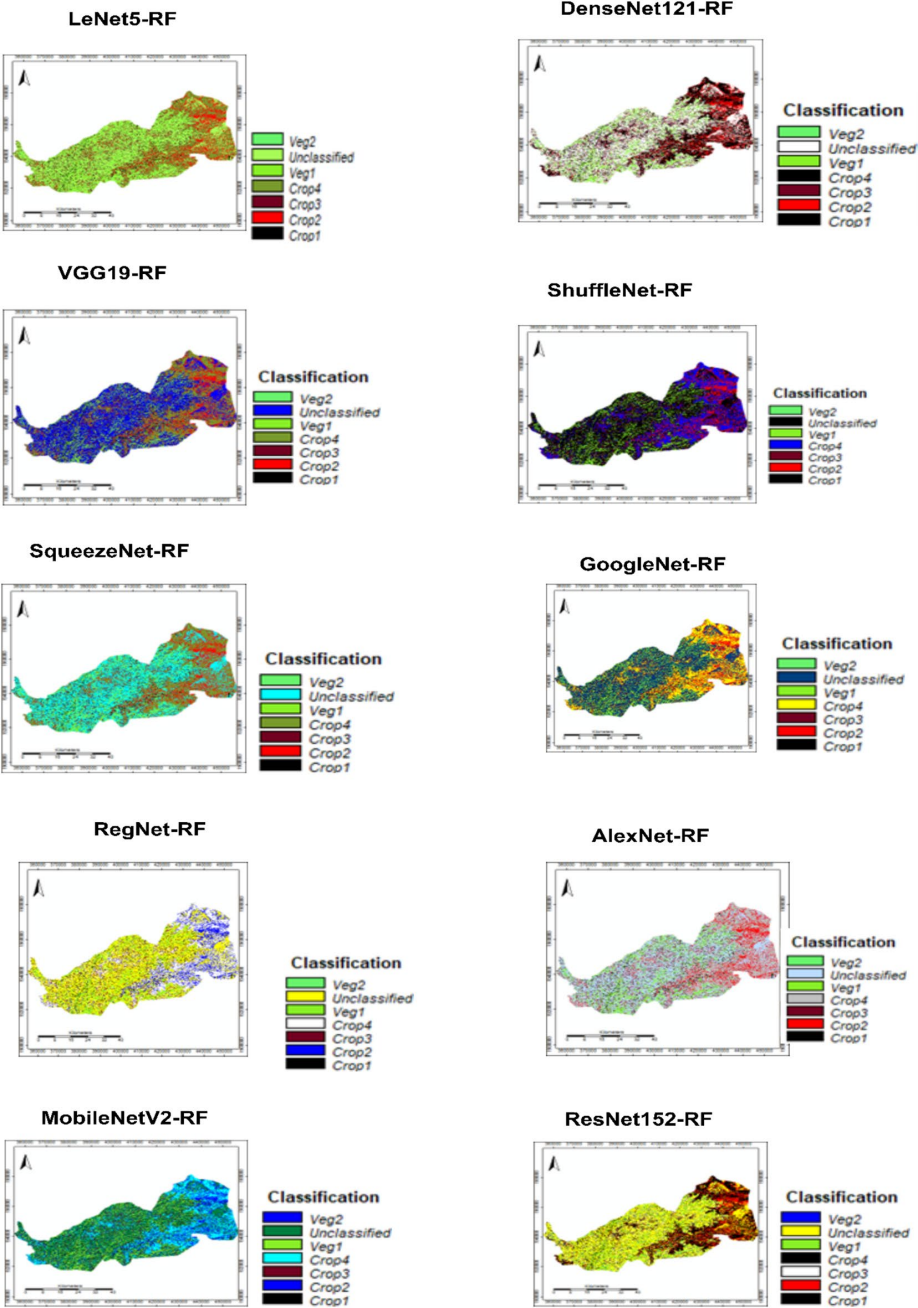
Results of land changes classification using a hybrid proposed system.

In Table 7, representing Group 2, a similar classification was performed using deeper architectures: ResNet152, AlexNet, GoogleNet, ShuffleNet, and DenseNet121, all fused with RF. The Built-up area peaks with GoogleNet-RF (66.83%), which is unusually high compared to other models. This could imply overfitting to urban patterns or confusion with high-reflectance surfaces such as bare soils or dry riverbeds. The Vegetation class is lowest in GoogleNet-RF (10.95%), reinforcing the assumption that this model may have misclassified vegetated areas as urban. Water bodies are best captured by ShuffleNet-RF (21.57%), which may be due to its efficiency in edge detection and differentiation of spectral water signatures. Bare Soil is highest in ResNet152-RF (22.00%), indicating that deeper networks with residual connections can better differentiate soil textures. The Other Land category again shows wide variability, lowest in GoogleNet-RF (3.69%), and highest in DenseNet121-RF (19.95%), reflecting model-dependent interpretations of mixed land cover types Fig. 6.

**Table 7:** Area and percentage of land changes for group 2 using the proposed Approach.

Models	ResNet152-RF		AlexNet-RF		GoogleNet-RF		ShuffleNet-RF		DenseNet121-RF	
**Classes**	**Area km²**	**Area %**	**Area km²**	**Area %**	**Area km²**	**Area %**	**Area km²**	**Area %**	**Area km²**	**Area %**
Built up	331.22	28.65%	450.37	38.93%	772.79	66.83%	336.5	29.10%	369.97	32.00%
Vegetation	283.35	24.51%	257.58	22.27%	126.52	10.95%	259.29	22.43%	286.22	24.77%
Water	122.55	10.60%	104.35	9.02%	115.47	9.99%	249.52	21.57%	171.64	14.85%
Bare Soil	254.35	22.00%	158.61	13.72%	98.84	8.54%	192.23	16.65%	97.63	8.44%
Other Land	164.68	14.25%	185.24	16.05%	42.53	3.69%	118.6	10.26%	230.69	19.95%
**Total**	**1**,**156.15**	100%	**1**,**156.15**	100%	**1**,**156.15**	100%	**1**,**156.14**	100%	**1**,**156.15**	100%

The potential suitability of the methods implemented in this study for land change classification in Najran City will be mentioned. Table 8 displays the differences between methods used in this research as below:

**Table 8:** Differences between CNN methods through the results of this work.

Num	Method	Results
1	LeNet5	Due to its limited depth, it was unsuitable for complex land change classification tasks.
2	VGG19	It was very suitable. It captured intricate features but was computationally expensive due to its depth. VGG19 is a simple and uniform architecture with small 3 × 3 convolutional filters.
3	SqueezeNet	It was suitable for land change classification in resource-constrained environments but sacrificed some accuracy. SqueezeNet was designed to be computationally efficient, using 1 × 1 convolutions to reduce model parameters.
4	RegNet	It offered flexibility regarding model size adapted to the available computational resources. RegNet focuses on providing efficient and scalable architectures using a set of design principles, including regularized networks.
5	MobileNetV2	It was suitable for land change classification tasks where computational resources are limited.
6	ResNet152	ResNet152 captured intricate features in land change but was computationally demanding. ResNet introduced residual connections, which help mitigate the vanishing gradient problem and enable training very deep networks.
7	AlexNet	It was suitable for land change classification tasks but is not as deep as recent architectures.
8	GoogleNet	It captured multi-scale features effectively, making it suitable for diverse land change patterns. GoogleNet introduced the inception module, utilizing different filter sizes simultaneously.
9	ShuffleNet	It was suitable for land change classification in resource-constrained environments. ShuffleNet focuses on reducing computational complexity by employing channel shuffling.
10	DenseNet121	It effectively captured dense features, potentially suitable for complex land change patterns. DenseNet connected each layer to every other layer in a feed-forward fashion, promoting feature reuse.

**Table 9:** Measures significant distinguish between architectures and computational complexity of CNN.

Num	Method	Depth	Parameters	Computational Complexity	Notable Features
1	LeNet5	Shallow	Moderate	Low	Early CNN, Handwritten digit recognition
2	VGG19	Deep	High	High	Small 3 × 33 × 3 filters
3	SqueezeNet	Moderate	Low-Moderate	Moderate	1 × 11 × 1 convolutions
4	RegNet	Variable	Variable	Variable	Scalable architecture
5	MobileNetV2	Moderate	Low	Moderate	Depth-wise separable convolutions
6	ResNet152	Very Deep	High	Moderate	Residual connections
7	AlexNet	Deep	Moderate	High	Winner of ImageNet 2012
8	GoogleNet	Deep	Moderate	High	Inception modules
9	ShuffleNet	Moderate	Low-Moderate	Low-Moderate	Group convolutions, channel shuffling
10	DenseNet121	Deep	High	Moderate	Dense connectivity

In comparing and evaluating the performance of various classifiers, it is essential to consider their architectural characteristics. LeNet5 is a shallow network with moderate parameters and low computational complexity, initially designed for handwritten digit recognition. VGG19, on the other hand, is a deep network with high parameters and computational complexity, distinguished by its use of small 3 × 3 filters. SqueezeNet features a moderate depth, low-moderate parameters, and computational complexity, emphasizing 1 × 1 convolutions for efficiency. RegNet offers a scalable architecture with variable depth, parameters, and computational complexity. MobileNetV2 is characterized by a moderate depth, low parameters, and moderate computational complexity, employing depth-wise separable convolutions. ResNet152, an intense network with high parameters, introduces residual connections to mitigate vanishing gradient problems. AlexNet, a deep network with moderate parameters and high computational complexity, won the ImageNet 2012 competition. GoogleNet, also deep with sensible parameters and computational complexity, incorporates inception modules. ShuffleNet, featuring reasonable depth, low-moderate parameters, and low-moderate computational complexity, employs group convolutions and channel shuffling. Finally, DenseNet121 is a deep network with high parameters and moderate computational complexity characterized by dense connectivity. The choice among these classifiers depends on specific use cases, considering factors like computational resources, model interpretability, and task requirements. Table 9 shows the differences in CNN model architectures in terms of computational complexity and their features.

### Confusion matrix analysis

A confusion matrix is a table that evaluates a classification model’s performance. The matrix is constructed by comparing the predicted and actual class labels. It helps identify the model’s errors, like false positives and negatives. Confusion matrices are a powerful tool for evaluating the performance of land change classification models. A confusion matrix identifies specific misclassification types, like confusion between land cover types. This information adjusts the model’s training data or improves the classification features. A confusion matrix helped identify the best-suited algorithm and was used to track changes in land use over time. This information assesses the effectiveness of land use and land management practices. Tables 10, 11, 12, 13, 14, 15, 16, 17, 18 and 19 show the confusion matrix resulting from evaluating the performance of the models proposed in this study.

The confusion matrices provided (Tables 10, 11, 12, 13, 14, 15, 16, 17, 18 and 19) offer a detailed breakdown of the performance of various terrain classification models used to classify different land types in Najran City. These matrices present the true vs. predicted class labels, which help evaluate the models’ classification performance by identifying errors like false positives and false negatives. A critical analysis of these matrices can reveal how well each model performed in differentiating between land cover types while also highlighting areas where the models struggled.

Table 10 shows that the LeNet5-RF model performs reasonably well in classifying the “Built-up” and “Vegetation” categories, with high values in the diagonal elements (61,200 for Built-up, 55,800 for Vegetation). However, Bare Soil and Other Land were moderately misclassified, as some 1,000 instances of Bare Soil were misclassified as Built-up. This issue with misclassifying water features saw 1,000 of the Water misclassified as Built-up, while 1,000 were misclassified as Vegetation. This suggests that the features might need some engineering adjustments to distinguish water bodies from built-up areas better.

**Table 10:** Confusion matrix generated by the LeNet5-RF model for terrain classification in Najran City.

Predicted \ Actual	Built-up	Vegetation	Water	Bare Soil	Other Land	Predicted
**Built-up**	61,200	1,500	1,000	1,000	1,000	65,700
**Vegetation**	1,500	55,800	1,000	1,000	1,000	60,300
**Water**	1,000	1,000	47,800	500	500	50,800
**Bare Soil**	1,000	1,000	500	20,700	1,000	24,200
**Other Land**	1,000	1,000	500	1,000	42,956.85	46,456.85
**Actual**	**68**,**866.67**	**62**,**828.89**	**53**,**753.33**	**23**,**237.78**	**48**,**235.56**	**256**,**922.22**

Table 11 highlights the impressive performance of the VGG19-RF model, especially for “Vegetation” and “Water,” where few misclassifications occur. There is an extremely low rate of misclassifications for “Built-up,” with 75,100 properly classified cases. Though strong overall, there are some misclassifications for “Vegetation” as “Bare Soil” (100), which could indicate a misalignment between certain vegetation and soil classes in the model’s training data.

**Table 11:** Confusion matrix generated by the VGG19-RF model for terrain classification in Najran City.

Predicted \ Actual	Built-up	Vegetation	Water	Bare Soil	Other Land	Total Predicted
**Built-up**	75,100	400	200	100	100	75,900
**Vegetation**	400	57,900	100	100	500	59,000
**Water**	200	100	43,000	50	50	43,400
**Bare Soil**	100	100	50	26,500	100	26,850
**Other Land**	100	500	50	100	48,154.53	48,904.53
**Total Actual**	**76**,**964.44**	**59**,**357.78**	**44**,**062.22**	**27**,**151.11**	**49**,**386.67**	**256**,**922.22**

Table 12 shows that the SqueezeNet-RF model performs well for most land types, particularly “Built-up” (75,300), and also performs well in classifying “Water” (35,500). Some challenges appear in classifying “Vegetation,” with a relatively high misclassification rate (3,000) predicted as “Built-up” and “Water” (500) misclassified as “Vegetation,” highlighting potential confusion between these land types.

**Table 12:** Confusion matrix generated by the SqueezeNet-RF model for terrain classification in Najran City.

Predicted \ Actual	Built-up	Vegetation	Water	Bare Soil	Other Land	Total Predicted
**Built-up**	75,300	3,000	500	500	500	79,800
**Vegetation**	3,000	56,200	500	500	1,000	61,200
**Water**	500	500	35,500	200	200	36,900
**Bare Soil**	500	500	200	21,700	1,500	24,400
**Other Land**	500	1,000	200	1,500	41,217.89	44,417.89
**Total Actual**	**84**,**131.11**	**62**,**746.67**	**39**,**622.22**	**24**,**255.56**	**46**,**166.67**	**256**,**922.22**

Table 13 shows that the RegNet-RF model classifies the “Built-up” and “Vegetation” classes well (46,600 and 58,300, respectively). This model is balanced, with no land types being too highly misclassified. A few wrong classifications occur in “Water” (200 misclassified as “Vegetation”) and “Bare Soil” (50 misclassified as “Water”), which possibly implies some difficulties in differentiating among these land categories.

**Table 13:** Confusion matrix generated by the RegNet-RF model for terrain classification in Najran City.

Predicted \ Actual	Built-up	Vegetation	Water	Bare Soil	Other Land	Total Predicted
**Built-up**	46,600	1,500	100	2,000	500	50,700
**Vegetation**	1,500	58,300	200	1,000	2,500	63,500
**Water**	100	200	7,900	50	100	8,350
**Bare Soil**	2,000	1,000	50	69,500	1,500	74,050
**Other Land**	500	2,500	100	1,500	50,368.36	54,968.36
**Total Actual**	**51**,**442.22**	**64**,**353.33**	**8**,**735.56**	**76**,**788.89**	**55**,**602.22**	**256**,**922.22**

Table 14 shows that the MobileNetV2-RF model renders high classification accuracies for “Built-up” (50,800) and “Vegetation” (32,100) and very low errors for other land types. Other than some errors from “Other Land” being confused with “Vegetation” (3,000) and “Bare Soil” (2,000), this points towards category confusion when features or patterns barely distinguish.

**Table 14:** Confusion matrix generated by the MobileNetV2-RF model for terrain classification in Najran City.

Predicted \ Actual	Built-up	Vegetation	Water	Bare Soil	Other Land	Total Predicted
**Built-up**	50,800	1,500	300	3,000	1,000	56,600
**Vegetation**	1,500	32,100	500	1,000	3,000	38,100
**Water**	300	500	17,800	200	300	19,100
**Bare Soil**	3,000	1,000	200	49,400	2,000	55,600
**Other Land**	1,000	3,000	300	2,000	78,124.91	84,424.91
**Total** **Actual**	**57**,**177.78**	**36**,**191.11**	**20**,**013.33**	**55**,**575.56**	**88**,**186.67**	**256**,**922.22**

Table 15 shows that the ResNet152-RF model exhibits excellent performance in the “Built-up” (67,900) and “Water” (25,100) categories, with few misclassifications for “Vegetation” and “Bare Soil.” On the other hand, misclassifications of “Water” as “Vegetation” (400) and “Bare Soil” as “Water” (200).

**Table 15:** Confusion matrix generated by the ResNet152-RF model for terrain classification in Najran City.

Predicted \ Actual	Built-up	Vegetation	Water	Bare Soil	Other Land	Total Predicted
**Built-up**	67,900	1,200	300	3,000	500	72,900
**Vegetation**	1,200	58,100	400	800	2,500	63,000
**Water**	300	400	25,100	200	100	26,100
**Bare Soil**	3,000	800	200	52,200	1,000	57,200
**Other Land**	500	2,500	100	1,000	33,736.44	37,836.44
**Total** **Actual**	**73**,**604.44**	**62**,**966.67**	**27**,**233.33**	**56**,**522.22**	**36**,**595.56**	**256**,**922.22**

Table 16 shows that the AlexNet-RF model performs well in the “Built-up” (94,700) and “Vegetation” (54,200) categories and has relatively low misclassifications everywhere. Somewhat more worrisome are the mingling confusions between “Water” (100) and “Built-up” (100), indicating that some challenges arise when distinguishing these classes in some contexts.

**Table 16:** Confusion matrix generated by the AlexNet-RF model for terrain classification in Najran City.

Predicted \ Actual	Built-up	Vegetation	Water	Bare Soil	Other Land	Total Predicted
**Built-up**	94,700	800	100	3,500	500	99,600
**Vegetation**	800	54,200	150	500	2,000	57,650
**Water**	100	150	21,900	50	100	22,300
**Bare Soil**	3,500	500	50	33,400	800	38,250
**Other Land**	500	2,000	100	800	38,900.40	42,300.40
**Total Actual**	**100**,**082.22**	**57**,**240.00**	**23**,**188.89**	**35**,**246.67**	**41**,**164.44**	**256**,**922.22**

Table 17 shows that the GoogleNet-RF model stands out in classifying “Built-up” (165,100), “Vegetation” (27,000), and “Water” (232.6) with very few misclassifications. The performance in “Bare Soil” and “Other Land” is commendable. Misclassification is relatively low, but a slight challenge is distinguishing “Water” from “Vegetation” (50 misclassified), which could arise due to visual similarities in water and vegetated areas in satellite images.

**Table 17:** Confusion matrix generated by the GoogleNet-RF model for terrain classification in Najran City.

Predicted \ Actual	Built-up	Vegetation	Water	Bare Soil	Other Land	Total Predicted
**Built-up**	165,100	300	100	4,500	200	170,200
**Vegetation**	300	27,000	50	100	500	27,950
**Water**	100	50	24,700	30	20	24,900
**Bare Soil**	4,500	100	30	21,100	100	25,830
**Other Land**	200	500	20	100	9,130.72	9,950.72
**Total Actual**	**171**,**731.11**	**28**,**115.56**	**25**,**660.00**	**21**,**964.44**	**9**,**451.11**	**256**,**922.22**

Table 18 shows how the ShuffleNet-RF classification is best for the classification of Built-Up: 64,800 and Vegetation: 49,900. It also has a fair accuracy for Water: 48,100, for example, whereas the model does not classify Other Land well and classifies it as Vegetation (500) and Bare Soil (800), which might stand for overlapping or non-distinctive features that separate land types of this class.

**Table 18:** Confusion matrix generated by the ShuffleNet-RF model for terrain classification in Najran City.

Predicted \ Actual	Built-up	Vegetation	Water	Bare Soil	Other Land	Total Predicted
**Built-up**	64,800	2,500	1,000	5,000	500	73,800
**Vegetation**	2,500	49,900	3,500	1,000	500	57,400
**Water**	1,000	3,500	48,100	1,500	300	54,400
**Bare Soil**	5,000	1,000	1,500	37,000	800	45,300
**Other Land**	500	500	300	800	22,871.75	24,971.75
**Total** **Actual**	**74**,**777.78**	**57**,**620.00**	**55**,**448.89**	**42**,**717.78**	**26**,**355.56**	**256**,**920.00**

Table 19 shows that DenseNet121-RF obtains good results for Built-up (76,000) and Vegetation (58,800). Its overall performance in classifying “Water” (35,200) is also notable. Minor misclassifications occur for “Water” (400 as “Built-up”) and “Bare Soil” (1,000 as “Other Land”), indicating some difficulty in distinguishing these classes clearly.

**Table 19:** Confusion matrix generated by the DenseNet121-RF model for terrain classification in Najran City.

Predicted \ Actual	Built-up	Vegetation	Water	Bare Soil	Other Land	Total Predicted
**Built-up**	76,000	1,200	400	3,500	500	81,600
**Vegetation**	1,200	58,800	600	800	2,500	63,900
**Water**	400	600	35,200	200	300	36,700
**Bare Soil**	3,500	800	200	20,000	1,000	25,500
**Other Land**	500	2,500	300	1,000	47,370.84	51,670.84
**Total Actual**	**82**,**215.56**	**63**,**604.44**	**38**,**142.22**	**21**,**695.56**	**51**,**264.44**	**256**,**922.22**

### Class-wise performance metrics

Table 20 summarizes key metrics across all models. VGG19-RF dominates with 97.56% accuracy, 98.91% precision, 98.92% recall, and 98.91% F1-score, achieving the most consistent and robust performance. GoogleNet -RF follows closely (96.15% accuracy), also displaying near-perfect results across all metrics, indicating minimal misclassification and highly reliable predictions. ShuffleNet-RF is the weakest performer (86.69% accuracy), likely affected by class confusion and lower generalization capability. GoogleNet-RF demonstrated the best balance with an F1-score of 99.05%, making it robust across all terrain classes. While VGG19-RF was slightly behind in raw F1 Score, it also delivered exceptional performance across all metrics (98.91% across the board), confirming its reliability. Compared to each other, GoogleNet-RF exhibited marginally better precision (99.06% vs. 98.92%), suggesting fewer false positives, while VGG19-RF held identical recall (98.91%). ResNet152-RF scored 92.26% accuracy, delivering strong, consistent classification across terrain types. AlexNet-RF closely followed with 94.26% accuracy, also showing stable performance with a 97.01% F1-score. DenseNet121-RF achieved 92.39% accuracy. RegNet-RF leads this group with 90.56% accuracy, but still trails behind the top-tier models by a narrow margin. SqueezeNet-RF and MobileNetV2-RF scored 94.13% and 93.58%, respectively, showing moderate effectiveness but with room for improvement. LeNet5-RF’s 88.92% accuracy is the lowest among deep models, potentially struggling with complex terrain distinctions.

**Table 20:** Performance results of the proposed models based on ACO algorithm for feature selection for terrain classification in Najran city.

Model	Accuracy	Precision	Recall	F1-Score
LeNet5-RF	88.92	92.76	92.47	92.51
VGG19-RF	97.56	98.92	98.91	98.91
SqueezeNet-RF	89.49	94.34	94.02	94.07
RegNet-RF	90.56	96.15	96.11	96.12
MobileNetV2-RF	88.83	93.84	93.46	93.5
ResNet152-RF	92.26	97.66	97.61	97.62
AlexNet-RF	94.26	97.03	97	97.01
GoogleNet-RF	96.15	95.83	96.47	96.15
ShuffleNet-RF	86.69	89.42	89.15	89.19
DenseNet121-RF	92.39	96.78	96.71	96.72

### Analysis of model architecture efficacy

The results directly link model performance to architectural design principles. The superior results from deeper, more complex networks such as VGG19, GoogleNet, and ResNet152 underscore their capacity to learn highly discriminative, hierarchical features from Landsat-8 imagery. The success of GoogleNet-RF, in particular, suggests that its inception modules, which capture multiscale features, are exceptionally well-suited to the diverse spatial patterns in land cover, from the fine textures of vegetation to the broad, homogeneous areas of bare soil.

Conversely, the lower accuracy of lightweight models such as MobileNetV2-RF and ShuffleNet-RF (OA: ~88–89%) highlights a clear trade-off between computational efficiency and classification power. While these models are designed for speed and lower parameter counts, this appears to come at the cost of their ability to capture the complex feature representations necessary for high-fidelity land cover discrimination in this specific context.

### Interpretation of results for sustainable land management

The high-accuracy classification results obtained from our hybrid CNN-RF framework provide more than just performance metrics; they yield actionable insights for sustainable land management. The following interpretation connects our quantitative findings to practical implications for different engineering and planning disciplines.

**For agricultural engineers and water resource managers**:

The analysis indicates that vegetated areas comprise approximately 14.08% to 25.04% of the study area, while bare soil measures 9.44% to 29.88%. This reversal pattern, as shown in various models such as RegNet-RF, suggests areas vulnerable to land degradation and soil exposure. With these accurate maps, agricultural engineers can identify locations for the strategic implementation of practices such as cover cropping and conservation tillage, which are strongly associated with reducing soil erosion. In addition, the accuracy of delineating water bodies (e.g., VGG19-RF water precision 98.9%) enables water engineers to achieve similar accuracy in monitoring surface water resources. This is particularly important when considering efficient irrigation designs and groundwater recharge zones in this arid environment, which supports the water conservation effort associated with Saudi Vision 2030.

**For urban planners and civil engineers**:

The built-up class was consistently identified, with areas ranging from 20.02% to 32.75%. The higher values from models like SqueezeNet-RF (32.75%) may indicate superior detection of low-density urban sprawl and peri-urban structures. This detailed spatial information on urban expansion is vital for civil engineers and urban planners. It enables quantification of encroachment into agricultural buffers, supports infrastructure planning, and provides a baseline for assessing the Urban Heat Island (UHI) effect. The confusion observed in some models between bright built-up surfaces and bare soil (e.g., in AlexNet-RF) underscores the need for the high-fidelity models we developed, like VGG19-RF, to avoid misallocating strategic planning resources.

**For environmental scientists and policy makers**:

The “Other Land” class, which captured transitional and mixed pixels (comprising 17.96% to 34.25% of the area), serves as an indicator of land-use instability and potential conversion. The high overall accuracy and Kappa coefficients (κ ≈ 0.97) of our top models provide policy-makers with a reliable and transparent evidence base. This reliability is crucial for enforcing land zoning regulations, monitoring the effectiveness of afforestation programs, and tracking progress towards sustainability targets. The robust performance of computationally efficient models like MobileNetV2-RF also indicates a pathway for developing near-real-time monitoring systems for ongoing environmental assessment.

### Comparative analysis of proposed systems based on feature selection algorithms: ACO vs. PSO Gf

To provide a comprehensive evaluation of our feature selection strategy, we implemented a Particle Swarm Optimization (PSO) algorithm as an alternative to our proposed Ant Colony Optimization approach. The comparative results between Table 19 (ACO) and Table 21 (PSO) reveal performance differences that warrant detailed analysis.

The PSO-optimized models demonstrate a consistent performance degradation across all architectures, with accuracy reductions ranging from 5.2% to 7.1%. For instance, the top-performing VGG19-RF model shows a decrease from 97.56% to 91.56% accuracy, while GoogleNet-RF declines from 96.15% to 90.15%. This pattern persists across precision, recall, and F1-score metrics, indicating that PSO’s feature selection strategy is less effective in identifying the most discriminative feature subsets from the CNN outputs.

The performance gap can be attributed to fundamental algorithmic differences. PSO, as a population-based optimizer operating in continuous space, tends to converge rapidly but may become trapped in local optima when dealing with high-dimensional, combinatorial feature selection problems. Its velocity-based update mechanism, while efficient for continuous optimization, is less well-suited to the discrete nature of feature subset selection. The algorithm’s tendency toward premature convergence often yields suboptimal feature combinations that fail to capture the intricate spectral-spatial relationships essential for accurate land cover classification.

In contrast, ACO’s probabilistic construction mechanism, combined with its pheromone-based positive feedback system, demonstrates superior capability in exploring the complex feature space. The stigmergic communication among artificial ants enables more effective navigation through the high-dimensional feature landscape, systematically reinforcing pathways that lead to high-quality feature subsets while maintaining diversity in the search process.

Furthermore, the performance disparity is most pronounced in deeper architectures like VGG19 and ResNet152, where the feature space is particularly complex. This suggests that ACO’s combinatorial optimization approach is better suited to handling the sophisticated feature hierarchies extracted by modern CNN architectures. The consistent superiority of ACO across all performance metrics validates its selection as the optimal feature selection strategy for our hybrid deep learning framework, ultimately contributing to the enhanced generalization capability and classification accuracy demonstrated in our final results.

**Table 21:** Performance results of the proposed models based on PSO for feature selection for terrain classification in Najran city.

Model	Accuracy (%)	Precision (%)	Recall (%)	F1-Score (%)
LeNet5-RF	82.92	86.76	86.47	86.51
VGG19-RF	91.56	92.92	92.91	92.91
SqueezeNet-RF	83.49	88.34	88.02	88.07
RegNet-RF	84.56	90.15	90.11	90.12
MobileNetV2-RF	82.83	87.84	87.46	87.5
ResNet152-RF	86.26	91.66	91.61	91.62
AlexNet-RF	88.26	91.03	91	91.01
GoogleNet-RF	90.15	89.83	90.47	90.15
ShuffleNet-RF	80.69	83.42	83.15	83.19
DenseNet121-RF	86.39	90.78	90.71	90.72

## Discussion and comparison of the methodology’s performance

This study implemented a hybrid deep learning-based technique for land cover classification, integrating various CNN architectures with an RF classifier. The consequences in Tables 5 and 6 imply that different CNN-RF hybrid fashions show varying performance throughout land cover categories. This highlights the importance of model choice based on the nature of the analyzed land cover. Additionally, we discovered that lightweight architectures, including MobileNetV2-RF and SqueezeNet-RF, offer efficient results, specifically for real-time, because of their decreased computational cost. Conversely, deeper architectures like ResNet152-RF and DenseNet121-RF presented progressive feature extraction results but required higher computational resources. The results propose a trade-off between accuracy and computational performance, essential for realistic land cover mapping applications.

Mohammed and team analyzed LULC changes in Selangor, Malaysia, using satellite imagery from 1991 to 2021. They employed SVM classification in ArcGIS and predicted future trends. Upasana et al. studied the Kamrup Metropolitan District in Northeast India over 22 years, using supervised machine learning for LULC analysis. They identified urban expansion, reduced cultivated land, and noted a growing Urban Heat Island effect. Sajjad et al. used remote sensing and GIS methods in Sahiwal District for over 40 years, employing MLC and correlating climate variations with NDVI. Ning et al. focused on the YRB, using the PLUS model to analyze landscape patterns and habitat quality evolution. Jan et al. classified Sentinel-2 data in Czech regions using RF on Google Earth Engine. Saeid et al. assessed LULC changes using Landsat time series and the RF classifier, exploring algorithmic impacts and auxiliary data. Zander et al. compared global LULC maps for accuracy. Sana et al. evaluated ArcGIS Pro and Google Earth Engine in Charlottetown, Canada. Eya et al. innovatively classified LULC in the Amazon basin using fusion methods. Katsuto et al. used Landsat time series and RF classifiers for mapping LULCC and forest disturbances in Vietnam. Ratnadeep et al. analyzed KMDA’s LULC dynamics over three decades. Using the CA-ANN model, Paulos et al. forecasted changes in the Upper Omo–Gibe River basin. Sergio et al. developed a fusion model for efficient land cover labeling based on RGB images. Kamran et al. enhanced remote sensing knowledge using Sentinel-2 bands and CNNs for semi-arid LULC classification in Pakistani cities.

The performance comparison revealed that deeper architectures like VGG19-RF and ResNet152-RF delivered superior classification accuracy (up to 97.56%), highlighting their capability in complex feature representation. VGG19-RF, in particular, demonstrated excellent generalization, minimal misclassification of key classes such as water and vegetation, and consistent sensitivity and specificity across all categories. GoogleNet-RF also showed strong results, especially in maintaining balanced precision and recall, making it suitable for practical agricultural and environmental monitoring applications.

The success of lightweight models such as MobileNetV2-RF and SqueezeNet-RF suggests potential for real-time or mobile applications, especially in regions with limited computational resources. However, their performance trade-offs were evident in distinguishing more complex terrain categories, indicating the continued relevance of deeper models for high-stakes decision-making in sustainable agriculture.

Existing studies have predominantly relied on standalone CNN models or conventional systems using classifiers. However, our hybrid CNN-RF approach balances feature extraction and classification accuracy, leveraging CNNs’ feature learning capabilities and RF’s robustness in handling class imbalances. This study’s results have exhibited superior performance compared to previous studies. The hybrid CNN and RF approach employed in this study for land change classification has been used. The robustness of our proposed method surpasses prior analyses, establishing a novel and effective hybrid system that demonstrates high accuracy in classification based on the results obtained.

This study’s findings are not only technically significant but also practically relevant. Accurate land cover and terrain classification in sustainable agriculture can support efficient water resource management, crop zoning, and desertification monitoring—key challenges for Najran City and other arid environments. The demonstrated methodology offers a replicable and adaptable framework for other regions facing similar ecological and agricultural constraints.

Recommendations include preprocessing with spectral indices like NDWI to resolve confusion between water and vegetation classes, and augmenting underrepresented classes like bare soil to mitigate class imbalance. Future research should consider integrating temporal satellite data to capture dynamic land changes and exploring ensemble models combining ACO with other metaheuristic optimizers.

The hybrid technique proposed in this study—uniting CNN-RF models with ACO optimization—offers a novel, accurate, and efficient framework for terrain classification and land cover mapping, with meaningful applications in promoting sustainable agriculture in water-scarce regions.

While this study presents a robust hybrid framework for LULC classification, several inherent limitations stemming from the methodological scope and data characteristics offer avenues for future research. The study’s spectral resolution is constrained by the bands available from the Landsat-8 OLI sensor. Although the selected bands are well-established for general LULC tasks, they may lack the granularity required to distinguish between specific crop types or subtle stages of vegetation stress with high confidence. The integration of hyperspectral data or the fusion of Sentinel-2’s red-edge bands, which are sensitive to chlorophyll content, could substantially improve the discriminative capacity for complex agricultural classes. The proposed system operates at a spatial resolution of 30 m, which is acceptable for regional-scale applications but can lead to mixed pixels in heterogeneous landscapes, particularly at the urban-agricultural interface. Future studies will evaluate the use of higher-resolution commercial imagery or leverage super-resolution algorithms to avoid mixed pixels and improve boundary delineation.

Addressing these limitations in future studies will improve the accuracy and robustness of the classification system and broaden its applicability across a range of operational contexts in precision agriculture and sustainable land management.

## Conclusion

This study proposed a hybrid framework combining deep CNNs, RF classifiers, and ACO for accurate land cover analysis and terrain classification in Najran City, Saudi Arabia. The integration of ACO effectively optimized feature selection, improving the performance of hybrid models by reducing redundancy and enhancing classification robustness. Among the ten CNN-RF hybrid models evaluated, VGG19-RF, GoogleNet-RF, and DenseNet121-RF demonstrated superior performance with high overall accuracy and Kappa Coefficients, particularly in identifying key land cover types such as vegetation, built-up areas, and water bodies. This confirms the potential of deep learning models to support large-scale land use analysis when combined with optimization techniques. The hybrid technique offers a powerful tool for mapping terrain characteristics vital to sustainable agriculture in semi-arid environments like Najran. Accurate terrain and land use classification provide essential input for agricultural planning, irrigation management, and land conservation strategies, contributing directly to long-term sustainability goals. This study advances methodological approaches in remote sensing and presents a practical solution to environmental monitoring and precision agriculture. Future work may explore temporal dynamics and integration with multi-sensor data for broader regional applications.

## Data Availability

Data supporting this work were obtained from the publicly available Internet at the following link: https://earthexplorer.usgs.gov/.
